# Physical performance and negative events in very old adults: a longitudinal study examining the *ilSIRENTE* cohort

**DOI:** 10.1007/s40520-024-02693-y

**Published:** 2024-02-12

**Authors:** Hélio José Coelho-Júnior, Riccardo Calvani, Alejandro Álvarez-Bustos, Matteo Tosato, Andrea Russo, Francesco Landi, Anna Picca, Emanuele Marzetti

**Affiliations:** 1 Department of Geriatrics, Orthopedics and Rheumatology, Università Cattolica del Sacro Cuore, Largo F. Vito 1, 00168 Rome, Italy; 2 Fondazione Policlinico Universitario “A. Gemelli” IRCCS, Largo A. Gemelli 8, 00168 Rome, Italy; 3 Biomedical Research Center Network for Frailty and Healthy Ageing (CIBERFES), Institute of Health Carlos III, Av. Monforte de Lemos 3−5, 28029 Madrid, Spain; 4 Department of Medicine and Surgery, LUM University, Str. Statale 100 Km 18, 70100 Casamassima, Italy

**Keywords:** Muscle strength, Muscle power, Mobility, Disability, Death, Aged

## Abstract

**Background:**

Declining physical performance in old age is associated with a wide range of negative health-related outcomes. However, it is unclear which physical capabilities should be prioritized to obtain prognostic information in older adults.

**Aims:**

To examine the associations between the performance on several physical function tests and falls, disability, and death in a well-characterized sample of very old Italian adults.

**Methods:**

This was a prospective cohort study of older adults who lived in the mountain community of the Sirente geographic area in Central Italy. Physical performance was assessed using isometric handgrip strength (IHG), walking speed (WS) at a usual and fast pace, 5-time sit-to-stand test (5STS), and sit-to-stand power measures. Appendicular skeletal muscle mass was estimated from calf circumference using a validated equation. History of falls, incident falls, and disability status according to basic Activities of Daily Living (ADLs) were recorded over two years. Survival status was obtained from the participants’ general practitioners and was confirmed by the National Death Registry over 10 years from enrolment. Linear, binary, and Cox regressions were performed to evaluate the association between physical performance measures and health outcomes.

**Results:**

The mean age of the 255 participants was 84.2 ± 5.1 years, and 161 (63.1%) were women.

Logistic regression indicated that IHG was significantly associated with incident ADL disability, whereas specific sit-to-stand muscle power was an independent predictor of death. No significant associations were observed between physical function and falls.

**Conclusions:**

Our findings indicate selective associations between physical function tests and the occurrence of negative events in very old adults, with poor IHG predicting disability and specific sit-to-stand muscle power being longitudinally associated with death.

**Supplementary Information:**

The online version contains supplementary material available at 10.1007/s40520-024-02693-y.

## Background

Physical function is a large construct that refers to voluntary motor actions that allow interaction between the individual and the environment [1]. After the third decade of life, physical function starts to deteriorate and reaches its lowest values in those 80 years of age and above [1, 2]. These age-related changes are associated with numerous negative events, such as reduced mobility, falls, institutionalization, and mortality [3, 4]. Furthermore, low performance on physical function tests is one criterion for identifying sarcopenia and physical frailty [5, 6], two highly prevalent and burdensome conditions of old age [7]. Therefore, it is recommended that healthcare providers who are in charge of the treatment of older adults carefully and actively check their patients’ physical function [5, 6].

Despite the importance of physical function in older adults, there is no agreement on the assessment tools that should be used in clinical practice and research. Isometric handgrip strength (IHG) and the 5-time sit-to-stand (5STS) tests, for example, are frequently considered comparable proxies of muscle strength [6]. However, this view has drawn criticism, given that IHG is weekly associated with muscle strength of other body districts and physical performance [8]. Furthermore, although walking speed (WS) provides information on physical and cognitive status as well as survival [9–11], it is typically assessed as a measure of disease severity or the sequelae of an acute illness (e.g., stroke) [6, 12].

Muscle power is an important physical function metric that pertains to the ability to generate muscle strength within a short time interval [13]. In the early 2000s, significant associations were found between lower-limb muscle power and mobility status in older adults [14]. Furthermore, lower-limb muscle power declines earlier and faster than other physical capacities [14–16] and is a better discriminator of physical independence, functional performance, and mobility than muscle strength [14–17].

The instruments and protocols used to measure muscle power in older adults vary considerably and involve the use of exercise machines or more complex instruments (e.g., isokinetic) [18, 19]. However, such methods may not provide a detailed evaluation of this physical capacity [18]. More recently, Alcalzar et al. [20, 21] validated easy-to-apply equations to estimate lower-limb muscle power parameters using the 5STS performance. In the validation study [21], authors showed that sit-to-stand muscle power was strongly associated with maximal muscle power recorded during leg press exercise using a linear position transducer. Moreover, sit-to-stand muscle power measures were more strongly associated with neuromuscular and cognitive function than 5STS performance.

Since then, studies have shown that sit-to-stand muscle power predicts many negative events in advanced age, including disability and hospitalization [17, 22] as well as mortality [22]. Comparisons among instruments and studies testing the predictive capacity of sit-to-stand muscle power against other physical function measures are necessary to clarify which physical capabilities should be prioritized for evaluation in older adults.

To expand the knowledge on the subject, the present study was conducted to examine associations between the performance on several physical function tests and falls, disability, and death in a thoroughly characterized sample of very old Italian adults.

## Methods

Data for the present investigation were gathered from the Aging and Longevity Study in the Sirente Geographic Area (ilSIRENTE) database [23]. ilSIRENTE was a prospective cohort study conducted in the mountain community of the Sirente geographic area (L’Aquila, Abruzzo) in Central Italy. The community living in this area is distributed in 13 towns and villages, all located at altitudes between 800 and 1400 m above sea level and surrounded by mountains. The Sirente area is mostly rural, with agriculture representing the main activity. The ilSIRENTE study was designed by the Department of Geriatrics of the Università Cattolica del Sacro Cuore (Rome, Italy) and the teaching nursing home Opera Santa Maria della Pace (Fontecchio, L’Aquila, Italy) in partnership with local administrators and primary care physicians of the Sirente Mountain Community Municipalities.

The study was conducted according to the principles of the Declaration of Helsinki and was approved by the Ethics Committee of the Università Cattolica del Sacro Cuore (Rome, Italy). Before enrolment, all participants or their proxies, when appropriate, provided signed informed consent.

### Study population

A list of all persons living in the Sirente area was obtained in October 2003 from the registry offices of the 13 municipalities included in the research. The identification of potential participants involved the selection of individuals who were born prior to 1 January 1924 and were living in that region during the initial survey period. Out of 429 individuals who were eligible for inclusion, 65 declined to partake, resulting in a final sample of 364 participants. For the present study, analyses were conducted in 255 individuals, after excluding those with a substantial physical disability (*n = *20) or cognitive impairment (*n = *14), incapacity to complete the physical performance tests (*n = *27), or with missing data (*n = *48) for the variables of interest. No formal sample size estimation was performed.

### Data collection

Baseline assessments began in December 2003 and were completed in September 2004. Clinical interviews and functional assessments were conducted at the study clinics in each town. Those who could not come to the study clinic because of physical or cognitive problems or transportation issues were assessed in their own homes. Follow-up visits took place after 24 months of baseline assessment. Information about medical history, medications, and lifestyle habits (e.g., smoking, alcohol consumption, physical activity) was collected using validated questionnaires [23]. All study procedures were conducted by trained personnel including specialized physicians, nurses, physiotherapists, medical residents, and medical students of the Department of Geriatrics of the Università Cattolica del Sacro Cuore, the teaching nursing home Opera Santa Maria della Pace, and primary care physicians. The principal investigator of the ilSIRENTE study (F.L.) is the custodian of the database.

Physical activity levels were estimated based on self-report. Participants indicated their patterns of physical activity in the last year according to the following options: (a) virtually no physical activity (bedridden or almost); (b) sitting most of the time, with brief periods of light walking or other light activities; (c) low-intensity activities (e.g., walking, dancing, fishing, hunting) at least 2−4 h a week; (d) moderate-intensity activities (e.g., running, uphill walking, swimming, gymnastics) at least 1−2 h a week or low-intensity activities more than 4 h a week; (e) moderate-intensity activities more than 3 h a week; (f) high-intensity activities most days of the week; and (g) walking more than 5 km a day at least 5 days per week. The concepts of low, moderate, and high intensity were explained to participants before data collection. Participants who reported options “e” to “g” were categorized as physically active. Alcohol abuse was operationalized as a daily consumption of more than 500 mL of wine (or an equivalent quantity of alcohol). Current smoking was operationally defined as the regular use of tobacco, with a minimum frequency of once per week, during the previous year.

The capacity to perform basic (ADL) and instrumental (IADL) activities of daily living was assessed using the subscale H of the Minimum Data Set for Home Care (MDS − HC) instrument [24]. The capacity to independently perform 10 ADLs (i.e., mobility in bed, transfer, locomotion in and outside home, dressing upper and lower body, eating, toilet use, personal hygiene, and bathing) in the last three days was scored as: (0) independent, (1) setup help only, (2) supervision, (3) limited assistance, (4) extensive assistance, (5) maximal assistance, and (6) total dependence. A score of 0 to 3 was attributed to the participant’s autonomy to perform seven IADLs (i.e., meal preparation, ordinary housework, managing finance, managing medication, phone use, shopping, transportation) in the last seven days, as follows: (0) independent; (1) some help; (2) full help; (3) performed by others. Both ADL and IADL were coded into an 8-category hierarchical scale, with 0 indicating total independence/capacity and 7 corresponding to total dependance/incapacity [25, 26]. Participants with an ADL score of 4 or greater were excluded from the analysis.

Multimorbidity was operationalized as the presence of two or more of the following conditions: obesity, coronary heart disease, cerebrovascular disease, congestive heart failure, peripheral artery disease, hypertension, lung disease (chronic obstructive pulmonary disease, emphysema, or asthma), osteoarthritis, diabetes, dementia, Parkinson’s disease, renal failure and cancer (non-melanoma skin cancer excluded). This operationalization has been widely used in the literature to define multimorbidity in older adults [27]. Clinical diagnoses were recorded using section J of the MDS − HC [24] based on self-report, information gathered from primary care physicians, performing physical examinations, and conducting comprehensive reviews of clinical documentation, including laboratory tests and imaging examinations.

Cognition was assessed by the cognitive performance scale (CPS). The CPS is generated from five items of the MDS − HC (level of consciousness, decision making, short-term memory, making self-understood, and eating performance) [28]. The items are integrated into a single, hierarchical cognitive rating scale encompassing seven categories that range from 0 (no cognitive impairment) to 6 (very severe cognitive impairment). Participants with CPS of 3 or above were excluded from the analysis.

Body height and weight were measured through a stadiometer and an analog medical scale, respectively. The body mass index (BMI) was calculated as the ratio between body weight (kg) and the square of height (m^2^). Calf circumference was taken on the dominant leg by measuring the largest girth (cm) between ankle and knee joints using an anthropometric tape while the participant was in a seated position. Values were rounded to the nearest 0.1 cm. Appendicular skeletal muscle mass (ASM) was estimated based on the equation developed by the COCONUT Study Group [29]:

${\text{ASM (kg)}}\hspace{0.17em}=\hspace{0.17em}-10.427+[\mathrm{calf circumference }({\text{cm}})\times 0.768]-({\text{age}}\times 0.029)+({\text{sex}}\times 7.523)$ where sex = 1 for men, sex = 0 for women.

IHG was measured once in each hand and the higher value (in kg) was used for analysis. The test was performed with participants sitting comfortably on a chair with their shoulders in a neutral position. The arm being assessed had the elbow flexed at 90º near the torso, and the hand neutral with thumb up. A maximal contraction was performed over four seconds using a handheld hydraulic dynamometer (North Coast Hydraulic Hand Dynamometer; North Coast Medical, Inc., Morgan Hill, CA, USA). WS was evaluated by measuring gait speed at usual and fast pace over a 4-m course, starting from a standing still position. The faster of two trials (in m/s) was used for the analysis. Participants were allowed to use walk devices if they needed. For the 5STS test, participants were requested to stand up and sit down from an armless chair with their arms folded across the chest five times as quickly as possible. The time needed to complete the task was recorded in seconds and used for the analysis. Absolute muscle power values were calculated according to the equation proposed by Alcazar et al. [20, 21]:

1 $$\mathrm{Absolute\, muscle\, power }\,({\text{W}})\hspace{0.17em}=\hspace{0.17em}\frac{\mathrm{Body\, weight }\left({\text{kg}}\right) \times 0.9 \times g \times [\mathrm{height\, }({\text{m}}) \times 0.5 -\mathrm{ chair\, height\, }({\text{m}})]}{[\frac{5\,\mathrm{STS\, test\, time }\,({\text{s}})}{{\text{no}}.\mathrm{ of\, STS\, repetitions}}] \times 0.5}$$

Relative (adjusted by body weight), allometric (adjusted by height squared), and specific (adjusted by ASM) muscle power values were subsequently calculated as follows:

2 $$\mathrm{Relative\, muscle\, power\, }({\text{W}}/{\text{kg}})\hspace{0.17em}=\hspace{0.17em}\frac{\mathrm{Absolute\, muscle\, power\, }({\text{W}})}{\mathrm{Body\, weight }({\text{kg}})}$$

3 $$\mathrm{Allometric\, muscle\, power }\,({\text{W}}/{{\text{m}}}^{2})\hspace{0.17em}=\hspace{0.17em}\frac{\mathrm{Absolute\, muscle\, power }\,({\text{W}})}{\mathrm{Height }\,({{\text{m}}}^{2})}$$

4 $$\mathrm{Specific\, muscle\, power }\,({\text{W}}/{\text{kg}})\hspace{0.17em}=\hspace{0.17em}\frac{\mathrm{Absolute\, muscle\, power }\,({\text{W}})}{\mathrm{ASM }\,({\text{kg}})}$$

Participants unable to complete the test as requested (e.g., with folded arms) were excluded from the analysis.

### Outcomes

#### Incident disability

Incident disability was operationalized as the development of incapacity to independently perform one or more ADLs, including dressing, eating, toilet use, bathing, mobility in bed, locomotion, and transfer at two-year follow-up.

#### Falls

Fall history and incident falls were assessed using item 5 of section K of the MDS − HC instrument [24]. Participants or their proxies were asked to report any fall event they had experienced during 90 days prior to baseline or follow-up visit.

#### Death

Survival status was obtained from the participants’ general practitioners and was confirmed by the National Death Registry [23]. Time to death was calculated from the date of the baseline visit to that of death. All events that occurred over 10 years from enrolment were included in the analysis.

### Statistical analysis

Continuous variables are expressed as mean ± standard deviation (SD), while categorical variables are reported as absolute numbers and percentages. Receiver operating characteristic (ROC) curve analyses were used to determine cutoff points of sit-to-stand muscle power equations. IHG (women < 16 kg, men < 27 kg), WS (< 0.9 m/s), and 5STS (> 15 s) were categorized according to the revised criteria of the European Working Group on Sarcopenia in Older People (EWGSOP2) [6]. However, there are no universally accepted cutoff points for physical function, with values varying according to the studied population [2, 30, 31]. Therefore, conducting the analysis exclusively with binary data might affect the results. On the other hand, continuous data allow associations between variables to be analyzed without categorizing participants according to an independent variable. Sensitivity and specificity were used to find the optimal cutoff values. Linear regressions were conducted to test the association between physical performance tests and history of falls (as continuous data using the number of falls in the last 90 days as the dependent variable). Poisson regressions were conducted to test the associations between physical performance tests, as binary data, and history of falls (as continuous data using the number of falls in the last 90 days as the dependent variable). Binary regression was conducted to test the associations between physical performance tests and incident (and historical) falls and disability. In this case, falls were analyzed as a binary variable. Analyses of falls were conducted using both continuous and binary data because age is associated with a high frequency of falls [32]. Therefore, a large share of older adults may experience fall events, thereby preventing comparisons between fallers and non-fallers. In contrast, the use of the number of falls (continuous) as a dependent variable identifies those who fall more often, potentially providing further information. Analyses of disability were focused on ADLs because basic activities are more closely related to mobility and are less influenced by cognitive factors than IADLs [33, 34]. Kaplan − Meier and Cox proportional hazards analyses were used to identify predictors of survival. Time until death was used as the “time” variable. The final models were adjusted for age, sex, BMI, physical activity levels, income, alcohol abuse, multimorbidity, self-reported health, history of falls (for incident falls), and unintentional weight loss. Physical function was used as an independent variable in all tests, and was analyzed both as a continuous and categorical variable. For all tests, the level of significance was set at 5% (*p* < 0.05). All *P*-values were determined by two-tailed tests. The SPSS software (version 23.0, SPSS Inc., Chicago, IL, USA) was used for all analyses.

## Results

Table 1 shows the main characteristics of the 255 study participants at baseline. Mean age was 84.2 years (± 5.1), and most participants were women (63.1%). Mean BMI values were within the normal range. Active smokers and participants with a history of alcohol abuse were 25.9% and 13.3%, respectively. Average IHG and 5STS values indicate that most participants had borderline low muscle strength and physical performance [35]. Mean WS performance was lower than cutoff values for sarcopenia [35]. Twenty participants reported at least one fall event within 90 days prior to enrolment. During the two-year follow-up, 20 (7.8%) participants fell and 37 (14.5%) became disabled in at least one ADL. Two-hundred fifty-one (98.4%) died within 10 years. Average survival from baseline was of 2626.7 ± 1717.0 days (approximately 87.5 months).

**Table 1:** Main characteristics of study participants (*n = *255)

Variables	
Age, years	84.2 ± 5.1
Sex (female), *n* (%)	161 (63.1)
Height, m	1.56 ± 0.8
Weight, kg	64.3 ± 11.7
BMI, kg/m^2^	26.0 ± 4.1
Appendicular skeletal muscle mass, kg	19.2 ± 5.1
Isometric handgrip strength, kg	33.4 ± 12.6
Walking speed at usual pace, m/s	0.61 ± 0.23
Walking speed at fast pace, m/s	0.79 ± 0.31
5-time sit-to-stand test, s	15.5 ± 6.7
Lower-limb muscle power	
Absolute, W	372.6 ± 119.0
Relative, W/kg	2.1 ± 0.8
Allometric, W/m^2^	56.2 ± 22.7
Specific, W/kg	9.1 ± 3.2
Current smoking, *n* (%)	17 (6.6)
Alcohol abuse, *n* (%)	34 (13.3)
Multimorbidity, *n* (%)	169 (66.1)
Physically active, *n* (%)	63 (24.7)
ADL score	1.4 ± 2.4
IADL score	3.0 ± 2.6
Self-rated health score	3.5 ± 0.8
Prior fall(s), n (%)	20 (7.8)

Data are shown as mean ± standard deviation and number (%)

ADL: activities of daily living; BMI: body mass index; IADL: instrumental activities of daily living

Physically active: performed moderate-intensity activities more than 3 h a week in the last year

### Cutoff points for sit-to-stand muscle power

Supplementary material (SM) 1 and 2 show ROC curves and the area under the curve (AUC) for sit-to-stand muscle power measures with falls and disability as outcomes. ROC curve analysis was performed to identify cutoff points for sit-to-stand muscle power measures based on the occurrence of negative events. Results indicate that sit-to-stand muscle power measures had a low AUC (< 0.6), which suggests that this test is a poor predictor of negative events in our population. The highest value was found for specific sit-to-stand muscle power and disability (0.4), and a cutoff value of 7.1 W/kg was identified considering a sensitivity of 0.80 and a specificity of 0.77.

The characteristics of participants with high and low specific sit-to-stand muscle power are shown in SM3. Participants with low specific sit-to-stand muscle power had worse performance in all functional tests.

### Physical function and falls

Table 2 shows the association between physical function and previous falls. In the unadjusted model, 5STS, WS at a normal and fast pace, and absolute, relative, allometric, and specific sit-to-stand muscle power were significantly associated with falls. However, significance was lost after accounting for covariates. No significant associations were observed between physical function tests and a history of falls in the logistic regression.

**Table 2:** Linear regression and logistic regression for the association between physical function (independent variable) and history of falls (dependent variable)

	Physical function (continuous)				Physical function (binary)			
	Unadjusted β (95% CI)	*P*-value	Adjusted β (95% CI)*	*P*-value	Unadjusted OR (95% CI)	*P*-value	Adjusted OR (95% CI)*	*P*-value
Isometric handgrip strength (kg)	− 0.003 (− 0.007, 0.001)	0.175	− 0.012 (− 0.027, 0.003)	0.128	0.957 (0.208, 4.398)	0.954	0.605 (0.115, 3.172)	0.552
Walking speed at usual pace (m/s)	** − 0.300 (− 0.473, − 0.128)**	**0.001**	0.067 (− 0.639, 0.773)	0.849	0.000 (0.000, 0.000)	0.998	0.000 (0.000, 0.000)	0.998
Walking speed at fast pace (m/s)	**− 0.317 (− 0.547, − 0.086)**	**0.007**	0.798 (− 0.063, 1.659)	0.068	–	–	–	–
5-time sit-to-stand test (s)	**0.018 (0.010, 0.025)**	**0.001**	− 0.028 (− 0.057, 0.001)	0.052	0.543 (0.217, 1.358)	0.192	0.577 (0.192, 1.732)	0.327
Absolute muscle power (W)	** − 0.001 (− 0.002, − 0.001)**	**0.016**	0.001 (− 0.003, 0.005)	0.609	–	–	–	–
Relative muscle power (W/kg)	** − 0.082 (− 0.144, − 0.020)**	**0.010**	0.169 (− 0.146, 0.483)	0.285	–	–	–	–
Allometric muscle power (W/m^2^)	** − 0.003 (− 0.005, − 0.001)**	**0.022**	0.005 (− 0.006, 0.016)	0.377	–	–	–	–
Specific muscle power (W/kg)	** − 0.021 (− 0.038, − 0.003)**	**0.020**	0.016 (− 0.068, 0.099)	0.707	1.398 (0.449, 4.351)	0.563	1.940 (0.502, 7.496)	0.337

OR: odds ratio; CI: confidence interval

^*^Adjusted for age, sex, body mass index, physical activity levels, income, alcohol abuse, multimorbidity, self-reported health, and unintentional weight loss

Bold denotes statistical significance

The relationship between physical function and the incidence of falls is shown in Table 3. Linear regression indicates that WS at a fast pace and absolute, relative, and allometric sit-to-stand muscle power measures were associated with the risk of falling. No significant associations were found in the fully adjusted model, logistic regression, or Poisson regression (SM4).

**Table 3:** Logistic regression for the association between physical function (independent variable) and incident falls (dependent variable)

	Physical function (continuous)				Physical function (binary)			
	Unadjusted OR (95% CI)	*P*-value	Adjusted OR (95% CI)*	*P*-value	Unadjusted OR (95% CI)	*P*-value	Adjusted OR (95% CI)*	*P*-value
Isometric handgrip strength (kg)	0.964 (0.926, 1.004)	0.080	0.113 (0.000, 0.000)	0.999	0.224 (0.047, 1.076)	0.062	0.494 (0.130, 1.880)	0.301
Walking speed at usual pace (m/s)	0.162 (0.020, 1.338)	0.091	0.000 (0.000, 0.000)	1.000	0.240 (0.031, 1.854)	0.171	0.249 (0.027, 2.298)	0.220
Walking speed at fast pace (m/s)	**0.120 (0.023, 0.632)**	**0.012**	0.000 (0.000, 0.000)	1.000	–	–	–	–
5-time sit-to-stand test (s)	**1.063 (1.000, 1.130)**	**0.051**	66.509 (0.000, 0.000)	0.997	0.512 (0.203, 1.294)	0.157	0.474 (0.147, 1.527)	0.211
Absolute muscle power (W)	**0.988 (0.979, 0.998)**	**0.018**	0.719 (0.0.00, 3.980)	0.999	–	–	–	–
Relative muscle power (W/kg)	**0.391 (0.191, 0.800)**	**0.010**	0.000 (0.000, 0.000)	0.998	–	–	–	–
Allometric muscle power (W/m^2^)	**0.968 (0.943, 0.995)**	**0.018**	0.383 (0.000, 4.18)	0.998	–	–	–	–
Specific muscle power (W/kg)	**0.918 (0.781, 1.079)**	**0.302**	0.003 (0.000, 0.000)	0.999	1.143 (0.362, 3.612)	0.820	0.615 (0.035, 10.881)	0.740

OR: odds ratio; CI: confidence interval

^*^Adjusted for age, sex, body mass index, physical activity levels, income, alcohol abuse, multimorbidity, self-reported health, and unintentional weight loss

Bold denotes statistical significance

### Physical function and disability

The association between physical function and incident disability is shown in Table 4. Negative and significant associations were observed between incident disability and 5STS, WS at a normal and fast pace, IHG of both hands, and absolute, relative, allometric, and specific sit-to-stand muscle power. No significant associations were found when results were adjusted for covariates.

**Table 4:** Logistic regression for the association between physical function (independent variable) and incident disability (dependent variable)

	Physical function (continuous)				Physical function (binary)			
	Unadjusted OR (95% CI)	*P*-value	Adjusted OR (95% CI)*	*P*-value	Unadjusted OR (95% CI)	*P*-value	Adjusted OR (95% CI)*	*P*-value
Isometric handgrip strength (kg)	**0.948 (0.917, 0.980)**	**0.002**	0.721 (0.115, 1.168)	0.184	**0.273 (0.096, 0.774)**	**0.015**	**0.216 (0.058, 0.808)**	**0.023**
Walking speed at usual pace (m/s)	0.121 (0.023, 0.636)	0.121	14.784 (0.003, 64,883.7)	0.529	0.558 (0.184, 1.692)	0.302	0.637 (0.176, 2.306)	0.492
Walking speed at fast pace (m/s)	**0.148 (0.040, 0.541)**	**0.004**	3.571 (0.001, 16,194.8)	0.767	–	–	–	–
5-time sit-to-stand test (s)	**1.067 (1.017, 1.120)**	**0.008**	0.753 (0.499, 1.135)	0.175	**0.414 (0.201, 0.852)**	**0.017**	0.627 (0.265, 1.488)	0.290
Absolute muscle power (W)	**0.989 (0.982, 0.997)**	**0.004**	1.006 (0.966, 1.048)	0.780	–	–	–	–
Relative muscle power (W/kg)	**0.511 (0.301, 0.866)**	**0.013**	1.157 (0.062, 21.697)	0.923	–	–	–	–
Allometric muscle power (W/m^2^)	**0.968 (0.949, 0.989)**	**0.002**	1.017 (0.919, 1.125)	0.742	–	–	–	–
Specific muscle power (W/kg)	**0.824 (0.717, 0.946)**	**0.006**	8.290 (0.000, 0.000)	0.963	**0.450 (0.205, 0.984)**	**0.045**	0.492 (0.196, 1.233)	0.130

OR: odds ratio; CI: confidence interval

^*^Adjusted for age, sex, body mass index, physical activity levels, income, alcohol abuse, multimorbidity, self-reported health, and unintentional weight loss

Bold denotes statistical significance

Results of logistic regression indicate that the occurrence of disability was significantly associated with 5STS, IHG, and specific sit-to-stand muscle power in the crude model. After adjusting the analysis for covariates, only IHG remained significantly associated.

### Physical function and death

The association between physical function tests and death is shown in Table 5. Kaplan−Meier analysis indicates that specific sit-to-stand muscle power and IHG were good predictors of death. In the Cox analysis, only specific sit-to-stand muscle power remained a significant predictor of death.

**Table 5:** Kaplan−Meier and Cox regression for the predictive capacity of physical function (independent variable) toward death (dependent variable)

	Kaplan–Meier (*χ*^2^)	*P*-value	Cox regression	*P*-value
Isometric handgrip strength (kg)	**5.333**	**0.021**	0.700 (0.432, 1.132)	0.146
Walking speed at usual pace (m/s)	0.287	0.592	0.816 (0.558, 1.195)	0.296
5-time sit-to-stand test (s)	0.726	0.394	0.902 (0.670, 1.216)	0.500
Specific muscle power (W/kg)	**7.973**	**0.005**	**0.709 (0.516, 0.974)**	**0.034**

Bold denotes significance

## Discussion

The main findings of the present study indicate that performance on physical function tests predicts negative events in very old adults. A low IHG was significantly associated with incident disability, while death was significantly predicted by low specific sit-to-stand muscle power. No significant associations were observed between prior or incident falls and physical performance tests.

Our results are at least partly supported by previous investigations that reported associations between IHG and disability in older adults. Lauretani et al. [14] found that Italian older adults with low IHG enrolled in the InCHIANTI study were more frequently mobility disabled. Similar results were found by Lopez-Teros et al. [36] and McGrath et al. [37] who examined the incidence of ADL disability in North American and Mexican older adults. In a study by Rantanen et al. [38], middle-aged and old men were followed up for ~ 25 years. Authors noted that the risk of disability increased linearly according to lower IHG tertiles [38].

In our study, only specific sit-to-stand muscle power significantly predicted death. Few investigations examined the association between muscle power measures and mortality [22, 39, 40]. Metter et al. [39] found that lower-limb muscle power, assessed using a 30-s Wingate test, was a significant predictor of death over 40 years in middle-aged men enrolled in the Baltimore Longitudinal Study of Aging. Fujita et al. [40] found that Japanese middle-aged men with low lower-limb muscle power estimated according to the vertical jump test had a two-fold greater risk of death. While the results of these studies were promising, it is worth noting that the instruments employed for muscle power assessment are typically used in sports settings. Therefore, their suitability for evaluating older adults, particularly those with mobility limitations, is questionable. Furthermore, those methods are not well-suited for practical, real-world settings. The study by Losa-Reyna et al. [22] was the first to examine the relationship between muscle power estimated according to the 5STS-based formula and mortality in older adults. Among almost 2000 old participants enrolled in the Toledo Study for Healthy Aging, authors found that men, but not women, with low relative sit-to-stand muscle power had an increased risk of death over a mean follow-up of 3.3 years [22].

A question that remains is why IHG and specific sit-to-stand muscle power, but not other physical performance tests, were significantly associated with incident disability and death. A possibility might be that the capacity of physical function parameters to predict negative events is affected by age [41]. Changes in WS performance, for instance, predict disability and death in middle-aged and “young” older adults over the mid and long term [9, 10]. However, in very old adults, the occurrence of negative events is more frequent [42], such that more specific instruments directly associated with the cause of the event might be necessary.

The completion of most ADLs requires grip strength. Dressing, grooming, and eating involve the manual control of instruments to be performed. Toilet use also requires grip strength to sit down and get up as well as to grab and manipulate toilet paper. Grip strength is also necessary for bed mobility to move from one side to another, sit down, and get up [43]. Furthermore, a low IHG may impede older adults from properly utilizing walking aids (e.g., cane, walker), thereby affecting mobility [44].

5STS, sit-to-stand muscle power measures, and WS at a fast pace require the activation of type II muscle fibers to sustain rapid movements [13]. The lack of ability of these physical capacities to predict death, except for specific sit-to-stand muscle power, suggests that the neuromuscular − endocrine axis might affect survival in the oldest old. Skeletal muscle mass decline has been associated with mortality [45, 46] also in those 80 years and older [41]. Besides its role in force generation, the skeletal muscle is an endocrine organ that communicates with other bodily systems through myokines, molecules with pluripotent effects that are synthesized and released by active skeletal myocytes [47–49]. Myokines influence important physiological processes at musculoskeletal, metabolic, neural, vascular, and immunological levels [49–52]. A reduced expression of several myokines has been found in muscle biopsies from critically ill patients [53], in whom a lower FNDC5/irisin ratio (precursor/myokine) has been independently associated with mortality. However, these assumptions are based on an isolated observation, not accompanied by the analysis of circulating myokines, and are therefore speculative.

Several investigations have reported that lower-limb muscle power is more strongly associated with mobility and disability than muscle strength [14–17]. Such findings led experts in the field to recommend this physical capacity be actively monitored and targeted in older adults [18, 54, 55]. Our results add to existing knowledge and reinforce recent recommendations by indicating that specific sit-to-stand muscle power, but not measures of muscle strength, is significantly associated with survival in the oldest old. These findings deserve consideration as they suggest that evaluation of sit-to-stand muscle power combined with ASM might provide additional information in comparison to standard physical function tests and should be routinely evaluated in older adults. Sit-to-stand muscle power equations require simple information (i.e., body weight, height, chair height) and are based on the results of an easy-to-apply field test, making it readily implementable in clinical practice [21]. Nevertheless, more studies are necessary to confirm our findings, given that cutoff values and age-related variations in muscle power show specific trajectories according to the studied population [17, 20, 31, 56, 57].

The findings that physical performance tests were not cross-sectionally or longitudinally associated with falls are not surprising. Other studies did not observe a significant association between physical performance tests and falls in older adults [58, 59]. There are some possible explanations for these results. Falls have a multifactorial nature. Risk factors for falls are commonly classified as intrinsic (e.g., age, sensory impairments) and extrinsic (e.g., home organization, footwear) [60]. According to Ambrose et al. [60], the risk of falls increases with the combination of these risk factors. In our study, we adjusted the analysis according to several possible intrinsic risk factors for falling, including, age, sex, BMI, previous falls, physical activity levels, and multimorbidity. However, no information on environmental factors was available. Another possible explanation is that the low prevalence and incidence of falls recorded did not allow significant differences to be observed between participants with high and low physical performance levels. A low prevalence of falls in older adults from rural areas has also been observed in other investigations [32, 61]. In the present study, participants had lower levels of disability at baseline and relatively good physical performance, according to the mean results of IHG and 5STS tests [6]. Such characteristics indicate that our sample was composed of very old people with fairly good health and relatively preserved physical performance, which might stem from long periods of manual labor and agricultural occupation as commonly observed in rural areas [62]. On the other hand, WS results were lower than cutoff values and only 24.7% of participants were physically active, suggesting that most older adults had mobility problems and spent their time in low-intensity activities or seated, which might reduce their risk of falls. Alternatively, the fact that balance was only indirectly measured through WS and 5STS suggests the possibility that different results could have been obtained if the static and dynamic balance were evaluated using recommended instruments [62]. Although mobility tests involve dynamic balance [62], specific instruments might be necessary to capture detailed information on balance performance [63]. However, the use of such instruments is problematic in epidemiological studies.

A major limitation of the present study is that cutoff points were determined and tested in the same population. This approach was chosen because the ilSIRENTE focuses on a very specific population, in which the use of cutoff points from different populations might produce inaccurate data. Indeed, studies reported different cutoff points for sit-to-stand muscle power in Brazilian, Italian, Colombian, and European older adults, indicating the relevance of geographical and social factors to these functional measures [31, 57, 64]. Further research examining a similar population is warranted to test the cutoff values proposed in the present study.

Other limitations of the present study must be acknowledged to allow better interpretation of our results. First, the time to falls and disability was not recorded, which impeded us from conducting survival analyses. Moreover, fall rates were not recorded over throughout 2 years. Second, ASM and muscle power were estimated according to calf circumference and indirect equations instead of recommended assessment tools [6]. This approach was chosen to allow the evaluation of participants both at the study sites and at home in a time-efficient manner. However, sit-to-stand muscle power received some criticism [65] and was only recently proposed. More studies on this method are necessary to provide further evidence on its validity. Third, information on the cause of death was not available, which prevented us from exploring the relationship between sit-to-stand muscle power measures and cause-specific mortality. Fourth, blood concentrations of myokines were not measured. This information is important because myokines may serve as a biomarker of muscle health [47, 66]. Fifth, the low occurrence of falls impeded from classifying participants in fallers and non-fallers. Sixth, physical activity was based on self-reported information, and the possibility that its assessment using performance-based measures (e.g., accelerometry) might have produced different results cannot be ruled out. Seventh, analyses were not adjusted according to the use of walking aids. Finally, we examined a cohort of relatively healthy very old adults who lived in a mountain region, and extrapolations to other populations should be made with caution.

## Conclusions and implications

Our findings indicate the existence of specific associations between physical function tests and the occurrence of negative events in very old adults, with poor IHG predicting disability and specific sit-to-stand muscle power being longitudinally associated with death. Although further studies are necessary to confirm our findings, these results suggest that upper-limb muscle strength, lower-limb muscle power, and muscle mass should be routinely monitored in advanced age to timely identify those at risk of negative events. The assessment of IHG, 5STS, and calf circumference requires only a few minutes and provides important information for the care of older adults.

### Supplementary Information

Below is the link to the electronic supplementary material.

Supplementary file1 (DOCX 63 KB)

Supplementary file2 (DOCX 31 KB)

Supplementary file3 (DOCX 16 KB)

Supplementary file4 (DOCX 15 KB)

## Data Availability

The datasets generated and analyzed during the current study are available from the corresponding author on reasonable request.
