# Supplementary material for: Physical performance and negative events in very old adults: a longitudinal study examining the ilSIRENTE cohort
Source: Aging Clin Exp Res. 2024 Feb 12;36(1):33. doi: 10.1007/s40520-024-02693-y (PMC10861604; doi:10.1007/s40520-024-02693-y)

**Supplementary Material 1**

Receiver operating characteristic (ROC) curves and area under the curve for muscle power measures (independent variable) and falls (outcome variable).

| **Table S1.** Area under the curve (AUC) for the association between muscle power measures and falls. | | |
| --- | --- | --- |
|  | **AUC** | **P-value** |
| Absolute muscle power (W) | 0.351 | 0.029 |
| Relative muscle power (W/kg) | 0.321 | 0.009 |
| Allometric muscle power (W/m^2^) | 0.341 | 0.020 |
| Specific muscle power (W/kg) | 0.422 | 0.252 |


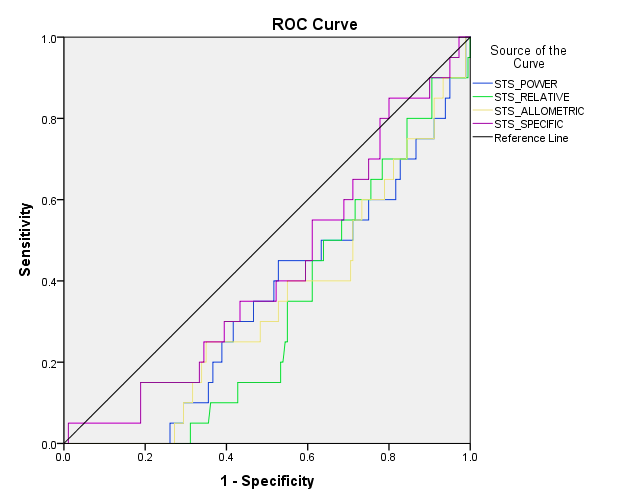

Supplement: Supplementary file 1 — Supplementary file1 (DOCX 63 KB) [file 40520_2024_2693_MOESM1_ESM.docx]
