# Supplementary material for: Physical performance and negative events in very old adults: a longitudinal study examining the ilSIRENTE cohort
Source: Aging Clin Exp Res. 2024 Feb 12;36(1):33. doi: 10.1007/s40520-024-02693-y (PMC10861604; doi:10.1007/s40520-024-02693-y)
Supplement: Supplementary file 3 — Supplementary file3 (DOCX 16 KB) [file 40520_2024_2693_MOESM3_ESM.docx]

**Supplementary Material 3**

| **Table S3.** Independent t-test for the main characteristics of study participants according to specific muscle power (n=255). | | |
| --- | --- | --- |
| Variables | **Low specific muscle power**  **(<7.1 W/kg, n=67)** | **High specific muscle power**  **(≥7.1 W/kg, n=188)** |
| Age, years | 81.7 ± 5.2 | 82.4 ± 5.1 |
| Sex (female), n (%) | 40 (15.7) | 114 (44.7) |
| Height, m | 152.6 ± 7.2 | 158.4 ± 8.8* |
| Weight, kg | 60.6 ± 11.5 | 65.3 ± 11.6* |
| BMI, kg/m^2^ | 26.0 ± 4.8 | 25.9 ± 3.8 |
| Appendicular skeletal muscle mass, kg | 15.9 ± 5.1 | 15.5 ± 4.5 |
| Isometric handgrip strength, kg | 26.6 ± 9.9 | 35.6 ± 12.7* |
| Walking speed at usual pace, m/s | 0.48 ± 0.20 | 0.66 ± 0.22* |
| Walking speed at fast pace, m/s | 0.60 ± 0.27 | 0.85 ± 0.30* |
| 5-time sit-to-stand test, s | 21.9 ± 9.1 | 13.1 ± 3.1* |
| Lower-limb muscle power |  |  |
| Absolute, W | 86.2 ± 33.1 | 160.2 ± 58.2* |
| Relative, W/kg | 1.41 ± 0.4 | 2.44 ± 0.8* |
| Allometric, W/m^2^ | 36.6 ± 13.1 | 63.0 ± 21.3* |
| Specific, W/kg | 5.4 ± 1.2 | 10.4 ± 2.7* |
| Current smoking, n (%) | 15 (5.9) | 49 (19.2) |
| Alcohol abuse, n (%) | 7 (2.7) | 26 (10.2) |
| Multimorbidity, n (%) | 23 (9.0) | 51 (20.0) |
| Physically active, n (%) | 5 (2.0) | 58 (22.7)* |
| ADL score | 2.0 ± 2.8 | 1.4 ± 2.3 |
| IADL score | 3.6 ± 2.6 | 3.1 ± 2.6 |
| Self-rated health score | 3.3 ± 0.7 | 3.5 ± 0.8 |
| Prior fall(s), n (%) | 4 (1.6) | 16 (6.3) |
| Data are shown as mean ± standard deviation and number (%). ADL: activities of daily living; BMI: body mass index; IADL: instrumental activities of daily living.  Physically active: performed moderate-intensity activities more than 3 hours a week in the last year.  *P<0.05 vs. low specific muscle power. | | |
