# Supplementary material for: Physical performance and negative events in very old adults: a longitudinal study examining the ilSIRENTE cohort
Source: Aging Clin Exp Res. 2024 Feb 12;36(1):33. doi: 10.1007/s40520-024-02693-y (PMC10861604; doi:10.1007/s40520-024-02693-y)
Supplement: Supplementary file 4 — Supplementary file4 (DOCX 15 KB) [file 40520_2024_2693_MOESM4_ESM.docx]

| **Table S4**. Poisson regression for the association between physical function (independent variable) and history of falls (dependent variable). | | | | |
| --- | --- | --- | --- | --- |
|  | **Unadjusted β (95% CI)** | **P-value** | **Adjusted β (95% CI)*** | **P-value** |
| Isometric handgrip strength (kg) | 0.60 (−1.11, 1.27) | 0.897 | 0.45 (−0.78, 1.69) | 0.470 |
| Walking speed at usual pace (m/s)^†^ | — | — | — | — |
| 5-time sit-to-stand (s) | 0.76 (0.03, 1.49) | 0.040 | 0.46 (−0.37, 1.30) | 0.278 |
| Sit-to-stand specific muscle power (W/kg) | 0.27 (−0.50, 1.06) | 0.488 | −0.02 (−0.92, 0.86) | 0.952 |
| CI: confidence interval.  *Adjusted for age, sex, body mass index, physical activity levels, income, alcohol abuse, multimorbidity, self-reported health, and unintentional weight loss.  ^†^ Hessian matrix singularity is caused by this parameter. | | | | |
